# Proteinuria Independently Predicts Unfavorable Outcome of Ischemic Stroke Patients Receiving Intravenous Thrombolysis

**DOI:** 10.1371/journal.pone.0080527

**Published:** 2013-11-22

**Authors:** Chih-Hao Chen, Sung-Chun Tang, Li-Kai Tsai, Shin-Joe Yeh, Kai-Hsiang Chen, Chen-Hua Li, Yu-Jen Hsiao, Yu-Wei Chen, Bak-Sau Yip, Jiann-Shing Jeng

**Affiliations:** 1 Stroke Center and Department of Neurology, National Taiwan University Hospital, Taipei, Taiwan; 2 Division of Neurology, Department of Internal Medicine, Far-Eastern Memorial Hospital, New Taipei City, Taiwan; 3 Department of Neurology, National Taiwan University Hospital Yun-Lin Branch, Yunlin, Taiwan; 4 Department of Neurology, National Taiwan University Hospital Hsin-Chu Branch, Hsinchu, Taiwan; 5 Department of Neurology, Landseed Hospital, Taoyuan, Taiwan; Harvard Medical School, United States of America

## Abstract

**Background and Purpose:**

Patients with low estimated glomerular filtration rate (eGFR) and proteinuria may be at increased risk for stroke. This study investigated whether low eGFR and proteinuria are outcome predictors in stroke patients treated with intravenous thrombolysis.

**Methods:**

We studied 432 consecutive stroke patients who received thrombolysis from January 2006 to December 2012, in Taiwan. Unfavorable outcome was defined as modified Rankin scale ≥2 at 3 months after stroke. Proteinuria was classified as negative or trace, mild, and moderate to severe. Using logistic regression analysis, we identified independent factors for unfavorable outcome after thrombolysis.

**Results:**

Of all patients, 32.7% had proteinuria. Patients with proteinuria were older, had higher frequencies of diabetes mellitus, hyperlipidemia, atrial fibrillation, lower eGFR, and greater severity of stroke upon admission than those without proteinuria. Proteinuria, not low eGFR, was an independent predictor for unfavorable outcome for stroke (OR = 2.00 for mild proteinuria, *p* = 0.035; OR = 2.54 for moderate to severe proteinuria, *p* = 0.035). However, no clear relationship was found between proteinuria and symptomatic hemorrhage after thrombolysis.

**Conclusions:**

Proteinuria is an independent predictor of unfavorable outcome for acute ischemic stroke in patients treated with intravenous thrombolysis, indicating the crucial role of chronic kidney disease on the effectiveness of thrombolysis.

## Introduction

Chronic kidney disease (CKD) is an important global public health issue [1]–[3]. Patients with CKD have a higher incidence of cardiovascular events, including ischemic stroke, than those without CKD [4], [5]. Impaired renal function is also associated with increased long-term mortality and poor outcome after stroke [6], [7]. Currently, intravenous thrombolysis remains the most effective and standard therapy for the acute ischemic stroke patient [8]–[10]. However, the impact of CKD on the functional outcome and hemorrhagic complications after thrombolysis remains inconclusive.

Two previous studies revealed that either increased serum creatinine level or reduced estimated glomerular filtration rate (eGFR), was associated with an unfavorable 3-month outcome in stroke patients receiving intravenous thrombolysis [11], [12] whereas another study did not find an association between eGFR<60 ml/min/m^2^ and poor functional outcome or death [13]. On the other hand, proteinuria (or albuminuria), an indicator of CKD, was found to be independently associated with increased risk of stroke and poor outcome after stroke [14]–[20]. One recent study shows that the presence of albuminuria after thrombolysis could be a predictor of hemorrhagic transformation in acute stroke patients [21]. The present study aimed to investigate whether low eGFR and proteinuria are independent predictors of the outcomes of acute ischemic stroke patients treated with intravenous thrombolysis in routine clinical practice.

## Methods

### Ethics Statement

This is a multi-center study including one medical center and three community hospitals in Taiwan. All participating hospitals have ongoing stoke registries which were approved by the National Taiwan University Hospital (NTUH) Research Ethics Committee (REC), NTUH Hsin-Chu Branch REC, NTUH Yun-Lin Branch REC, and Landseed Institutional Review Board, to prospectively collect information on acute stroke patients, including initial stroke severity, risk factors, stroke mechanisms, and outcome. All patients gave their written informed consent.

### Patients

This study reviewed consecutive ischemic stroke patients receiving intravenous (IV) recombinant tissue plasminogen activator (rt-PA) from January 2006 to December 2012. Patients were treated for up to 4.5 hours after stroke onset [8], [22]. Demographic data, and vascular risk factors including hypertension, diabetes mellitus, dyslipidemia, atrial fibrillation, coronary artery disease, prior stroke, and smoking were recorded. Initial laboratory results, including a complete blood count, coagulation testing, glucose, liver, and renal function, were also recorded. A follow-up brain image as computed tomography or magnetic resonance image was routinely performed 24 to 36 hours after IV rt-PA to determine any occurrence of intracerebral hemorrhage (ICH). The stroke subtype was defined according to the Trial of Org 10172 in Acute Stroke Treatment (TOAST) classification [23].

Our study subjects received IV rt-PA dosage that ranged from 0.6 to 0.9 mg/kg, and the standard dosage was defined as 0.9 mg/kg. Based on the Taiwan Thrombolytic Therapy for Acute Ischemic Stroke (TTT-AIS) study, the standard-dose of rt-PA may not be optimal for treating patients with old age and/or severe stroke due to increased risk of symptomatic ICH and mortality [24]. Therefore, a lower dosage (around 0.6 to 0.7 mg/kg) may have been chosen based on the patient's age and stroke severity.

### Determination of proteinuria and impaired renal function

Urine for urinalysis was collected in every patient on admission. If the patient had two or more urinalysis results in the admission course, the one most close to the stroke onset was used. The level of urinary protein was examined using a urine dipstick and was classified into negative (dipstick reading of −), trace (+/−), or positive (1+ to 4+). We categorized the dipstick results into two groups in regard to proteinuria: absence (negative and trace results); or presence (1+ to 4+). The severity of proteinuria was sub-classified as mild (1+, approximately 20 mg/dL to 70 mg/dL) or moderate to severe (2+ to 4+, ≥75 mg/dL). The serum creatinine level was obtained at admission. An estimated glomerular filtration rate (eGFR) was calculated using the Modification of Diet in Renal Disease (MDRD) equation: eGFR = 186.3×(serum creatinine)^−1.154^×(age)^−0.203^×(0.742 if the subject is female) [25]. We categorized the eGFR results into 3 groups: eGFR ≥60, 45–59, and <45 mL/min/1.73 m^2^ of body surface area.

### Outcome

The functional status at 3 months was defined by modified Rankin Scale (mRS) scores. Unfavorable outcome was defined as mRS ≥2. A hemorrhagic event was defined by the evidence of any ICH on the head images within the initial 36 hours. Symptomatic hemorrhage was defined as a neurological deterioration (NIHSS ≥4 points) within 36 hours with no radiological findings that might have been responsible for this deterioration other than a hemorrhage.

### Statistical Analysis

The clinical characteristics according to hospital level (medical center versus community hospitals), functional outcome, and proteinuria status were compared using the Fisher's exact test, chi-squared test, Student's t-test, and Mann-Whitney *U*-test with relevant variables as indicated. The multivariable logistic regression analysis models were used with unfavorable outcome (mRS ≥2) and hemorrhagic transformation event as the dependent variables. The independent variables entered in the analyses were age, sex, initial NIHSS score, severity of proteinuria, and level of eGFR. Statistical test results were considered significant if *p*<0.05. Statistical analysis was performed using the _SPSS_ software package version 20.0 (SPSS Inc., Chicago, IL, USA).

## Results

From January 2008 to December 2012, there were 432 consecutive acute ischemic stroke patients receiving IV rt-PA. The mean±SD age of the study subjects was 67.2±12.2 years and 263 patients (60.9%) were male. The mean±SD serum creatinine level was 1.17±0.59 mg/dL (102.9±52.7 µmol/L), and eGFR of 68.4±22.7 mL/min/m^2^. One hundred and fifty-five patients (36.0%) had an eGFR<60 mL/min/m^2^. The median initial NIHSS score was 13. Regarding the dosage administered, 227 patients (52.5%) received a relatively low dose (0.6 to 0.7 mg/kg) of rt-PA whereas the remaining patients received a dose of >0.7 mg/kg of rt-PA. The incidence of hemorrhagic transformation was 22.7%, and the symptomatic ICH was 5.9%. The proportion of patients with a favorable outcome at 3 months was 34.0%. In addition, the NIHSS on admission, functional outcome, and hemorrhagic complications were similar between the medical center and community hospitals (Table S1).

Urinalysis data were available for 404 patients. There were 132 patients (32.7%) with at least 1+ urinary protein as shown on the urine dipstick exam who were categorized into the proteinuria group. These patients were elderly and had a relatively high prevalence of diabetes mellitus, dyslipidemia, atrial fibrillation, and initial NIHSS score than patients without proteinuria (Table 1). Unfavorable outcome for stroke (mRS ≥2) was more frequently seen in patients with proteinuria than in those without (78.8% vs. 59.9%, *P*<0.001) and the 3-month mortality rate was slightly higher in the proteinuria group (9.1% vs. 5.5%, *p* = 0.18). The occurrence of hemorrhagic transformation was slightly more common in patients with proteinuria (26.5% vs. 21.0%, *p* = 0.21), whereas the incidence of symptomatic hemorrhage was not different between the two groups (5.9% vs. 6.1%, *p* = 0.94).

**Table 1:** Demographics and clinical characteristics of the study patients.

	Patients without proteinuria (n = 272)	Patients with proteinuria (n = 132)	*P* value
Age, years	66.4±12.4	69.9±11.4	0.01
Male	160 (58.8)	81 (61.4)	0.63
Body mass index, kg/m^2^	24.9±3.9	24.9±3.7	0.98
*Stroke risk factors*			
Hypertension	193 (71.2)	104 (78.8)	0.11
Diabetes mellitus	71 (26.3)	51 (38.6)	0.01
Dyslipidemia	84 (31.0)	53 (40.8)	0.05
Atrial fibrillation	115 (42.4)	73 (55.3)	0.02
Coronary artery disease	45 (16.6)	31 (23.5)	0.10
Prior stroke	60 (22.1)	26 (19.7)	0.57
Smoking	67 (24.7)	28 (21.2)	0.44
*Initial clinical characteristics*			
Systolic blood pressure, mmHg	159.9±29.4	164.3±34.5	0.19
Diastolic blood pressure, mmHg	90.3±19.1	91.1±22.5	0.74
Serum creatinine, µmol/L	92.1±25.6	126.8±82.2	<0.001
Blood ureanitrogen, mmol/L	6.21±2.27	8.17±4.16	<0.001
eGFR, ml/min/m^2^	72.6±19.6	59.1±26.0	<0.001
Blood glucose, mmol/L	6.98±2.43	7.56±2.83	0.05
HbA1c, %	6.2±1.4	6.5±1.3	0.02
Total cholesterol, mmol/L	4.54±0.99	4.48±1.04	0.60
Proteinuria level			
Negative	216 (79.4)	0 (0)	<0.001
Trace (+/−)	56 (20.6)	0 (0)	
Mild (1+)	0 (0)	75 (56.8)	
Moderate to severe (≥2+)	0 (0)	57 (43.2)	
NIHSS on admission (IQR)	12 (8-18)	15 (9-20)	0.01
rt-PA administration			
Dosage, mg/kg	0.73±0.14	0.70±0.14	0.11
Higher dose (>0.7 mg/kg)	131 (48.2)	51 (38.6)	0.07
Time to treatment, min (IQR)	131 (100–160)	135 (105–160)	0.35
*Stroke subtype*			
Cardioembolism	113 (41.5)	77 (58.3)	0.001
Large-artery atherosclerosis	71 (26.1)	33 (25.0)	
Small-vessel occlusion	34 (12.5)	7 (5.3)	
Others	54 (19.9)	13 (9.8)	

Values are number (percentage), or mean (±standard deviation) except for NIHSS and time to treatment [median (IQR)].

NIHSS indicates National Institutes of Health stroke Scale; IQR, interquartile range.

In univariate analysis for an unfavorable outcome (mRS≥2), age, sex, presence of atrial fibrillation, presence of proteinuria, and higher NIHSS score were identified as significant factors (*p*<0.05, Table S2). Other renal function indicators including the factor of eGFR<60 ml/min/m^2^ were not significantly related to an unfavorable outcome. In the multivariate logistic regression analysis, the presence of proteinuria remained a significant risk factor, with higher urinary protein levels corresponding to a higher risk of unfavorable outcome (OR = 2.00 for mild proteinuria, 95% CI = 1.05–3.81, *p* = 0.035; OR = 2.54 for moderate to severe proteinuria, 95% CI = 1.07–6.02, *p* = 0.035; Table 2). Mild proteinuria was associated with a two-fold increased risk of a hemorrhagic event after rt-PA use in both unadjusted and adjusted models, while severe proteinuria did not show such an association (Table 3). On the other hand, the severity of reduced eGFR showed no significant impact on functional outcome and hemorrhagic complications.

**Table 2:** Multivariate analysis of unfavorable outcome.

	Unadjusted OR (95% CI)	*P* value	Adjusted OR (95% CI)*	*P* value
Proteinuria level				
Negative or trace	1.00		1.00	
Mild	2.12 (1.18 – 3.79)	0.012	2.00 (1.05-3.81)	0.035
Moderate to severe	3.14 (1.52 – 6.49)	0.002	2.54 (1.07-6.02)	0.035
eGFR level (mL/min/1.73 m^2^)				
≥60	1.00		1.00	
45 – 59	1.46 (0.86 – 2.48)		1.02 (0.56-1.88)	0.948
<45	1.53 (0.83 – 2.82)	0.236	0.93 (0.43-2.03)	0.858

* , adjusted for age, sex, initial NIHSS, and atrial fibrillation. In the multivariate model for the proteinuria level, the eGFR level was additionally adjusted. In the multivariate model for the eGFR level, the proteinuria level was additionally adjusted.

Unfavorable outcome was defined as modified Rankin scale ≥2 at 3 months after stroke.

**Table 3:** Association between the severity of proteinuria and hemorrhagic transformation after thrombolysis.

	Unadjusted OR (95% CI)	*P* value	Adjusted OR (95% CI)*	*P* value
All hemorrhage				
Proteinuria level				
Negative or trace	1.00		1.00	
Mild	2.12 (1.22 – 3.69)	0.008	1.95 (1.10 – 3.46)	0.023
Moderate to severe	0.62 (0.28 – 1.37)	0.236	0.43 (0.18 – 1.04)	0.062
Symptomatic hemorrhage				
Proteinuria level				
Negative or trace	1.00		1.00	
Mild	1.14 (0.41 – 3.23)	0.801	1.07 (0.37 – 3.08)	0.902
Moderate to severe	0.89 (0.25 – 3.16)	0.855	0.70 (0.18 – 2.81)	0.617

* , adjusted for age, sex, initial NIHSS, atrial fibrillation, and eGFR level.

## Discussion

The main finding of our study was that in acute ischemic stroke patients treated with IV rt-PA, proteinuria was strongly related to unfavorable outcome at 3 months, and the effect remained significant even after adjusting for age, gender, and stroke severity. In addition, its detrimental effect was increased proportionally to the level of proteinuria. In contrast, we found that reduced eGFR, based on the creatinine level at admission, was not a predictor of poor functional outcome or hemorrhagic complications.

According to previously published reports, several prognostic factors were found consistently to be associated with poor outcome after rt-PA administration, such as older age, higher NIHSS score at baseline, current infarction on baseline imaging scans, high blood glucose, higher blood pressure, or premorbid functional deficits [26]–[29]. However, impaired renal function was seldom discussed among these studies. Theoretically, CKD patients are known to have abnormalities in coagulation and platelet function that favor thrombosis in the untreated state and then augment bleeding risks in the setting of antiplatelet drugs and anticoagulants [30]. Although CKD itself is not a contraindication to intravenous rt-PA administration in major clinical trials or guidelines [22], [31], [32], it is not known whether CKD patients can achieve equally good functional outcomes or worse bleeding complications as compared to patients without CKD.

One Switzerland study involving 196 ischemic stroke patients treated with rt-PA reported that a high initial serum creatinine level was independently associated with a poor outcome (defined as mRS score ≥3) at 3 months [11]. Another multi-center stroke registry involving 578 patients receiving rt-PA in Japan found that an eGFR<60 mL/min/m^2^ was an independent predictor toward ICH, poor outcome at 3 months, and mortality [12]. However, a report with 74 patients treated with rt-PA in a stroke center in the United States showed that an eGFR<60 mL/min/m^2^ was not associated with increased ICH, poor functional outcome, or death [13].

In our study, the commonly used indicators for renal dysfunction such as serum creatinine, blood urea nitrogen, or eGFR level were not different between favorable and unfavorable outcome groups. An eGFR<60 mL/min/m^2^ was not a predictor of unfavorable outcome or hemorrhagic transformation. As demonstrated by a large population-based study, the graded risk of cardiovascular events and mortality rose sharply for subjects with an eGFR<45 mL/min/m^2^ [4]. However in our study, even though eGFR<45 mL/min/m^2^ was used as a cut-off point for impaired renal function, eGFR was still was not associated with an unfavorable outcome for ischemic stroke.

Proteinuria is increasingly being recognized as an indicator and as a screening tool for CKD [14], [25]. Proteinuria is a sign of persistent dysfunction of the glomerular barrier and often precedes any detectable decline in renal filtration function [14]. Proteinuria itself is associated with approximately 50 to 70% increased risk of stroke [16], [18]. A large-scale stroke registry in Japan reported that proteinuria independently conferred increased risks of neurologic deterioration, mortality, and poor functional outcome among patients with acute ischemic stroke [20]. One recent single center study in Korea demonstrated that the presence of micro- and macro-albuminuria after intravenous thrombolysis was associated with hemorrhagic transformation, including parenchymal hemorrhage and symptomatic ICH [21]. To our knowledge, the present study is the first report to demonstrate that proteinuria is independently attributed to unfavorable outcome in patients treated with rt-PA, and the effect seems to be correlated to the severity of proteinuria.

The exact mechanism by which proteinuria is independently associated with unfavorable outcome after rt-PA-treated ischemic stroke is unclear. First, the presence of albuminuria is a predictor of hemorrhagic transformation after ischemic stroke, including those treated with intravenous thrombolysis [21], [37]. In our study, a mild degree of proteinuria (i.e., 1+ on dipstick exam, approximately 20–70 mg/dL) was associated with a two-fold increased risk of a hemorrhagic event, which might be one explanation for the unfavorable outcome in this group of patients. However, the net effect of proteinuria on symptomatic hemorrhage in our study was null, so other mechanisms might be involved in the pathogenesis of an unfavorable outcome. One currently favored mechanistic explanation is that proteinuria (or albuminuria) is a marker of generalized endothelial dysfunction [33], [34]. A greater capillary permeability for albumin in the systemic vasculature may lead to a generalized hemodynamic strain and disequilibrium, which ultimately initiates atherosclerosis. Furthermore, albuminuria is related to elevated serum concentrations of von Willebrand factor and other markers of endothelial dysfunction, a factor that might contribute to the formation of microthromboses [35], [36]. In our study, patients with proteinuria had more severe neurological deficits at stroke onset, which might reflect an earlier and global atherosclerosis process. Therefore, the presence of proteinuria might correlate more strongly to the overall vascular burden and atherosclerotic state than eGFR itself. In addition, whether the existence of proteinuria would weaken the efficacy of rt-PA in vascular recanalization in acute stroke patients deserves further evaluation. Taken together, these could be the reasons why proteinuria, but not eGFR, is independently associated with an unfavorable outcome.

There are some limitations of our study. First, not every patient receiving thrombolytic therapy had a urine dipstick examination, resulting in a slightly smaller sample size than initially expected. Even though, we found that proteinuria was still more important than eGFR in predicting functional outcome of thrombolytic patients at 3 months after stroke if we only analyzed the patients receiving both eGFR and urine examination (404 of 432 patients). Second, eGFR and proteinuria were determined by creatinine levels and urinary dipstick results at admission, which may have been affected by acute stroke effects. The possibility of transient proteinuria cannot be excluded. A pre-stroke evaluation and even repeated urinary examination would be desirable to abate the influence of stroke. Third, the urinary dipstick analyses were quasi-quantitative, and not standardized in our participating hospitals. Although the dipstick test is cost-effective and simple, its sensitivity is not always high enough since it is useful only for urinary protein >300 to 500 mg/day. Thus, it would more precise to evaluate the presence of micro albuminuria or urinary albumin-to-creatinine ratio.

In summary, our study demonstrated that proteinuria was an independent predictor of an unfavorable outcome after rt-PA treatment of acute ischemic stroke patients. A further prospective study is indicated to delineate the precise relationship between proteinuria or micro albuminuria and stroke outcome after rt-PA administration. In patients with proteinuria, further randomized controlled trials might be warranted to see if additional therapeutic strategies to reduce proteinuria would improve the efficacy of thrombolysis.

## Supporting Information

Table S1 **Demographics comparison between medical center and community Hospitals.**
(DOCX)

Table S2 **Univariate analysis of unfavorable outcome.**
(DOCX)
