# Supplementary material for: Proteinuria Independently Predicts Unfavorable Outcome of Ischemic Stroke Patients Receiving Intravenous Thrombolysis
Source: PLoS One. 2013 Nov 22;8(11):e80527. doi: 10.1371/journal.pone.0080527 (PMC3838417; doi:10.1371/journal.pone.0080527)
Supplement: Table S1 — Demographics comparison between medical center and community Hospitals. (DOCX) [file pone.0080527.s001.docx]

**Table S1.** Demographics comparison between medical center and community Hospitals

|  | Total (n = 432) | Medical Center (n = 281) | Community Hospitals (n = 151) | *P* value |
| --- | --- | --- | --- | --- |
| Age, years | 67.2 ± 12.2 | 68.3 ± 12.4 | 65.0 ± 11.6 | 0.01 |
| Male | 263 (60.9) | 169 (60.1) | 94 (62.3) | 0.67 |
| Body mass index, kg/m^2^ | 24.9 ± 3.8 | 24.7 ± 3.5 | 25.2 ± 4.2 | 0.26 |
| *Stroke risk factors* | | | | |
| Hypertension | 316 (73.3) | 201 (71.5) | 115 (76.7) | 0.25 |
| Diabetes mellitus | 128 (29.8) | 90 (32.0) | 38 (25.7) | 0.17 |
| Dyslipidemia | 152 (35.6) | 98 (35.0) | 54 (36.7) | 0.72 |
| Atrial fibrillation | 191 (44.3) | 145 (51.6) | 46 (30.7) | <0.001 |
| Coronary artery disease | 77 (17.9) | 50 (17.8) | 27 (18.0) | 0.96 |
| Prior stroke | 88 (20.4) | 65 (23.1) | 23 (15.3) | 0.06 |
| Smoking | 106 (24.6) | 58 (20.6) | 48 (32.0) | 0.01 |
| NIHSS on admission (IQR) | 13 (8－19) | 14 (8－19) | 11 (8－19) | 0.46 |
| Unfavorable outcome | 285 (66.0) | 179 (63.7) | 106 (70.2) | 0.17 |
| *Hemorrhagic transformation* | | | | |
| Any hemorrhage | 100 (23.1) | 72 (25.6) | 28 (18.5) | 0.10 |
| Symptomatic hemorrhage | 27 (6.2) | 16 (5.7) | 11 (7.3) | 0.52 |

Unfavorable outcome was defined as modified Rankin ≥2 at 3 months after stroke.
