# Supplementary material for: Proteinuria Independently Predicts Unfavorable Outcome of Ischemic Stroke Patients Receiving Intravenous Thrombolysis
Source: PLoS One. 2013 Nov 22;8(11):e80527. doi: 10.1371/journal.pone.0080527 (PMC3838417; doi:10.1371/journal.pone.0080527)
Supplement: Table S2 — Univariate analysis of unfavorable outcome. (DOCX) [file pone.0080527.s002.docx]

**Table S2.** Univariate analysis of unfavorable outcome

|  | Favorable outcome (n = 147) | Unfavorable  outcome (n = 285) | *P* values |
| --- | --- | --- | --- |
| Age, years | 64.1 ± 11.8 | 68.7 ± 12.1 | <0.001 |
| Male | 104 (70.7) | 159 (55.8) | 0.003 |
| Body mass index, kg/m^2^ | 25.4 ± 3.5 | 24.6 ± 3.9 | 0.06 |
| *Stroke risk factors* | | | |
| Hypertension | 102 (69.9) | 214 (75.1) | 0.25 |
| Diabetes mellitus | 46(31.5) | 82 (29.0) | 0.59 |
| Dyslipidemia | 52 (35.6) | 100 (35.6) | 0.99 |
| Atrial fibrillation | 51 (34.9) | 140 (49.1) | 0.005 |
| Coronary artery disease | 20 (13.7) | 57 (20.0) | 0.11 |
| Prior stroke | 27 (18.5) | 61 (21.4) | 0.48 |
| Smoking | 41 (28.1) | 65 (22.8) | 0.23 |
| *Clinical parameters upon admission* | | | |
| Systolic blood pressure, mmHg | 162.1 ± 29.4 | 161.3 ± 32.3 | 0.82 |
| Diastolic blood pressure, mmHg | 91.1 ± 17.9 | 91.2 ± 21.4 | 0.95 |
| Serum creatinine, µmol/L | 105.1 ± 61.0 | 101.7 ± 47.9 | 0.53 |
| Blood ureanitrogen, mmol/L | 6.40 ± 2.87 | 7.00 ± 3.21 | 0.06 |
| eGFR, ml/min/m^2^ | 69.7 ± 21.8 | 67.8 ± 23.2 | 0.42 |
| eGFR< 60 ml/min/m^2^ | 46 (31.3) | 110 (38.7) | 0.13 |
| Proteinuria (n=404) | 28 (20.4) | 104 (39.0) | <0.001 |
| Blood glucose, mmol/L | 7.17± 2.58 | 7.27± 2.69 | 0.74 |
| HbA1c, % | 6.4 ± 1.4 | 6.3 ± 1.4 | 0.44 |
| Total cholesterol, mmol/L | 4.53 ± 0.93 | 4.53 ± 1.03 | 0.99 |
| NIHSS on admission (IQR) | 8 (6 – 13) | 16 (10 – 21) | <0.001 |
| < 10 | 87 (59.2) | 60 (21.1) |  |
| 10 - 19 | 46 (31.3) | 141 (49.5) |  |
| ≥ 20 | 14 (9.5) | 84 (29.5) |  |
| Higher rt-PA dose (>0.7 mg/kg) | 79 (53.7) | 126 (44.2) | 0.06 |
| Time to treatment, min (IQR) | 130 (96 – 153) | 134 (106 – 163) | 0.09 |
| *Stroke subtype* | | | |
| Cardioembolism | 50 (34.0) | 142 (49.8) | <0.001 |
| Large-artery atherosclerosis | 21 (14.3) | 88 (30.9) |  |
| Small-vessel occlusion | 35 (23.8) | 16 (5.6) |  |
| Others | 39 (26.5) | 35 (12.3) |  |
